# Effect of Spheroidal Age on Sorafenib Diffusivity and Toxicity in a 3D HepG2 Spheroid Model

**DOI:** 10.1038/s41598-019-41273-3

**Published:** 2019-03-19

**Authors:** Christoph Eilenberger, Mario Rothbauer, Eva-Kathrin Ehmoser, Peter Ertl, Seta Küpcü

**Affiliations:** 1 Institute of Applied Synthetic Chemistry and Institute of Chemical Technologies and Analytics, Faculty of Technical Chemistry, Vienna University of Technology, Getreidemarkt 9, 1060 Vienna, Austria; 2 Institute of Synthetic Bioarchitectures, Department of Nanobiotechnology, University of Natural Resources and Life Sciences, Vienna, Muthgasse 11, 1190 Vienna, Austria

## Abstract

The enhanced predictive power of 3D multi-cellular spheroids in comparison to conventional monolayer cultures makes them a promising drug screening tool. However, clinical translation for pharmacology and toxicology is lagging its technological progression. Even though spheroids show a biological complexity resembling native tissue, standardization and validation of drug screening protocols are influenced by continuously changing physiological parameters during spheroid formation. Such cellular heterogeneities impede the comparability of drug efficacy studies and toxicological screenings. In this paper, we demonstrated that aside from already well-established physiological parameters, spheroidal age is an additional critical parameter that impacts drug diffusivity and toxicity in 3D cell culture models. HepG2 spheroids were generated and maintained on a self-assembled ultra-low attachment nanobiointerface and characterized regarding time-dependent changes in morphology, functionality as well as anti-cancer drug resistance. We demonstrated that spheroidal aging directly influences drug response due to the evolution of spheroid micro-structure and organo-typic functions, that alter inward diffusion, thus drug uptake.

## Introduction

Despite the growing number of available anti-cancer drugs and various management regimes, some cancer types still remain without effective treatment strategies^1^. One of these cancer types is hepatocellular carcinoma (HCC), which is currently the second leading cause of cancer-related death with over 800.000 new cases diagnosed worldwide^2^. Even though, radiotherapy, resection, liver transplantation, and systemic chemotherapy represent the state-of-the-art treatment^3^, 60% of patients are still relapsing after surgery due to the aggressiveness of hepatocellular carcinoma^4,5^. This high incident of cancer recurrence demands the development of novel and more effective anti-cancer drugs. During the drug development process cytotoxicity tests based on conventional two-dimensional (2D) *in vitro* cell-based followed by *in vivo* animal models and clinical trials are routinely performed to assess the efficacy of novel drug candidates^6^. Despite a large number of early drug candidates, only 10% of compounds progress successfully through clinical phases, with a high prevalence of drug failures at late-stage clinical trials, thus generating enormous expenses before discontinuation^7^. One reason for this unsatisfactory situation is based on the inability to reliable identify promising candidates for use in early-stage clinical trials^8^. It is generally accepted that the majority of drug failures in later stages are in part caused by overestimation of data derived from 2D *in vitro* cell culture tests, where the unnatural cellular microenvironment leads to alterations in drug response levels^9^.

To overcome these drawbacks, one promising strategy is based on the establishment of three-dimensional (3D) cell cultures such as multi-cellular spheroids. These *in vivo*-like cell aggregates are surrounded by natural extra-cellular matrix (ECM) that promote direct cell-cell interaction, and thus recapitulate structures and functions of the native organs and tissue^10–12^. In cancer research, multi-cellular spheroids can be used to simulate intact human tumors featuring similar tissue architectures that are composed of cells of different phenotypes including proliferating, non-proliferating and necrotic subpopulations^13,14^. Since multi-cellular spheroids based on human-derived cells display adequate chemical and physical parameters influencing cell biology such as oxygen tension, compactness, apoptosis inhibition^15^, damage repair^16^, and permeability^17^, they are also a good candidate to replace animal testing due to their improved predictive capability^18^. Any reduction of animal tests is not only ethically desirable but would also reduce one of the main cost-drivers in drug development process^19^. For these reasons, multi-cellular spheroids are extensively used as promising *in vitro* models for evaluating therapeutic anti-cancer strategies including chemotherapy^20,21^, antibody-based immunotherapy^22^, gene therapy^23^ and combinatorial therapies^24^.

Despite the many advantages of multi-cellular spheroids over monolayer cultures^25–28^, some limitations still prevent the integration of 3D cell culture models into mainstream drug discovery pipelines. For instance, the lack of standardization in cell culture protocols often leads to variations in structure and composition of the established multi-cellular spheroids, all known to heavily affect the outcome of drug delivery and efficacy studies^29^. It is important to note that the selected culturing method significantly influences spheroid size, shape, density, surface topography and microstructure that may alter their behavior^30^. In addition to variations in structure, multi-cellular spheroids also comprise of cell populations in different proliferative stages including proliferation, quiescence and apoptosis, which leads to heterogeneous cell responses during chemical and physical treatments, thus making the comparability of drug exposure studies a difficult task^31,32^.

To ensure reproducible generation of multi-cellular spheroids and to increase the reliability of *in vitro* 3D-cell based assays, a set of quality parameters including area, perimeter, solidity and roundness have been introduced to increase the reproducibility of toxicity tests, efficacy studies and drug penetration assessments^33^. Although the benefits of these quality parameters in multi-cellular spheroid cultures are well established, the influence of spheroid cultivation time, also referred to as spheroidal age, on dose-response relationships in drug screening studies still remains an underestimated factor. The present work sets out to provide a better understanding how spheroidal age influences the outcome of drug screening studies using multi-cellular spheroids. In the present work, we specifically investigate how spheroidal age modulates diffusivity, resistance and toxicity of sorafenib, an FDA-approved multi-kinase inhibitor against liver cancer. Our 3D hepatocellular carcinoma spheroid model is generated using a novel protein-based nanobiointerface that reliably eliminates cell-surface interactions over long periods of time. Our self-assembled nanobiointerface is based on the S-layer protein SbpA derived from *Lysinibacillus sphearicus* CCM 2177 and exhibits outstanding cell-repulsive and anti-fouling properties^34–36^, thus effectively promoting the formation 3D HepG2 spheroids in microwells without the need of any external forces. We show that spheroid quality remains constant over 18 days in culture in the presence of SbpA-coated protein surfaces, while ultrastructural morphology and organo-specific metabolic evaluations are used to differentiate between early-stage, mid-stage and late-stage spheroids. Following the identification of spheroidal ages, drug diffusivity, toxicity and resistance are determined in an attempt to describe the interplay between spheroidal age and efficacy of drugs when employing *in vitro* 3D cell culture models.

## Materials and Methods

### S-layer Coating of Microwell Plates

Proteins were isolated from *Lysinibacillus sphaericus* CCM 2177 (SbpA) and subsequently purified as reported elsewhere^37^. To reconstitute the S-layer protein solution, 5 mg of lyophilized protein was dissolved in guanidine hydrochloride (5 M in 50 mM Tris-(hydroxymethyl)aminomethane x HCl buffer, pH 7.2) and dialyzed against Milli-Q-water (Millipore, Austria) for 1 h at 4 °C. Afterward, the protein solution was centrifuged at 1300 rpm for 15 min at 4 °C to remove self- assembly products. The concentration was adjusted to 100 μg/mL in a recrystallization buffer (0.5 mM Tris-(hydroxymethyl)aminomethane, 10 mM CaCl_2_, pH 9), 250 μL of the solution were added to U-bottom 96-well tissue culture plates (Greiner-Bio-One, Austria) and incubated over night at room temperature.

### Cell Culture and Spheroid Generation

Hepatocellular carcinoma cells (HepG2, HB-8065, ATCC, USA) were cultivated in minimal essential medium (MEM, Sigma-Aldrich, Austria) supplemented with 10% fetal bovine serum (FBS, Gibco, USA), 1% GlutaMax^TM^ (Life Technologies, Thermo Fisher Scientific, USA) and 1% antibiotic/antimycotic solution (Sigma-Aldrich, Austria). The cells were cultivated in 75 cm^2^ cell culture flasks at 37 °C in 5% CO_2_ humidified atmosphere as adherent monolayers. For spheroid generation, trypsinized cells were pelleted at 1250 rpm for 5 min (Megastar 1.6R, VWR) prior homogenization through a syringe to separate larger cell clusters into individual cells. Cells were seeded at an initial cell concentration of 10.000, 5.000 and 3.000 cells per well in SbpA-coated 96-well microtiter plates (Greiner Bio-one, Germany) and centrifuged at 1250 rpm for 10 minutes.

### Albumin and Urea Secretion

Spheroid medium supernatants of day 1, 3, 6, 9, 12, 15 and 18 were collected, centrifuged at 1250 rpm for 10 minutes to remove cell debris and stored at −20 °C until sample analysis. The amount of human serum albumin (HSA) secreted into the culture medium was determined by Human Albumin ELISA Kit (Abcam, UK) according to the manufacturer’s protocol. For ELISA, the supernatant was diluted 1:100 in dilution buffer. Urea was measured using a colorimetric assay kit from BioVision (Germany) according to the manufacturer’s protocol.

### Cytochrome P_450_ 3A4 and metabolic activity

HepG2 spheroids at different incubation times (1, 3, 6, 9, 12, 15 and 18 days) were washed with 1X phosphate buffered saline (PBS; Sigma-Aldrich, Austria), 50 µL of 3 µM P450-Glo™ substrate (Promega, Germany) were added to individual microtissues and incubated for 1 hour at 37 °C, 5% CO_2_. Then, 25 µL of substrate medium were transferred to a 96-white plate and CYP 3A4 activity was measured according to the manufacturer’s protocol. The remaining microtissues were used to quantify spheroid viability by CellTiter-Glo^®^ 3D Cell Viability Assay (Promega, Germany).

### Transmission Electron Microscopy (TEM)

Spheroids were fixed in 4% formaldehyde solution (Carl Roth, Austria) at 4 °C and embedded in agarose (2% in Caco buffer) as previously reported^38^. The ultrathin sections were observed with a transmission electron microscope (FEI Tecnai G2 20, FEI, Netherlands) operating at 120 kV and images were acquired with an FEI Eagle 4 K camera (Nikon, Japan).

### Histology

HepG2 spheroids were washed with 1X PBS (Sigma-Aldrich, Austria) after 3, 6, 12 and 18 days of incubation fixed with 4% paraformaldehyde in PBS (pH 7.6) at 4 °C and stored in PBS. For histological analysis, the HepG2 spheroids were cut in 3‐μm serial sections, deparaffinized in xylene and rehydrated in a graded alcohol series. Histological staining was performed with hematoxylin and eosin (H&E).

For hypoxia-induced-factor-1α (HIF-1α) immunohistochemistry staining, rehydrated sections were placed in a rack filled with 10 mM sodium citrate buffer pH 6.0 (Sigma-Aldrich, Austria) and heated at 100 °C for 20 minutes by a vegetable steamer for antigen retrieval. Protein blocking was conducted by 5% BSA in 10X Tris-buffered saline pH 7.6 (TBS; Sigma-Aldrich, Austria) for 1 hour and incubated with primary anti-HIF-1α mAb (1:50; Abcam, Germany) at 4 °C overnight. Samples were washed 3X with TBS, followed by incubation with secondary antibody Alexa Fluor^®^ 488 (1:200; Abcam, Germany) for one hour at room temperature. Nuclei were counterstained with DAPI (1:1000; Thermo-Fischer, Austria). Bright field images were acquired by bright field microscope (IX71, Olympus, Germany) equipped with a digital camera (XC10, Olympus, Germany) and Olympus IX 83 Live-cell microscope using DAPI (Ex: 350/Em: 470) and GFP (Ex: 488/Em: 519) fluorescence bandpass filters.

### Drug Screening

For monolayer culture, HepG2 cells were seeded at 3.000 cells per well in 96-well flat bottom plates and incubated for 6 days. Prior drug exposure, the monolayer reached a confluency of approximately 80%. For 3D culture, S-layer coated 96-well U-bottom plates were seeded at a cell density of 3.000 cells per well and incubated for 6 days. Drug exposure was carried out with different concentrations of sorafenib diluted in DMSO. Cells were treated with different concentrations of sorafenib (0–100 µM) in triplicates and incubated for 24 and 48 hours at 37 °C and 5% CO_2_. For analysis, AlamarBlue^®^ reagent (Invitrogen, Austria) was added directly to medium (10% v/v) and incubated overnight at 37 °C. Fluorescence was measured at ex/em 560/595 nm and absorbance was measured at 565 nm and 595 nm (Infinite F200, Tecan, Austria). The cytotoxicity index was determined using the untreated cells as a negative control and the IC_50_ was extrapolated from the dose-response graph. For dose-age responses, HepG2 spheroids were treated with 100 µM sorafenib for 24 h up to 12 days of incubation.

### Quantitative Live/Dead assay

For determination of spheroid viability, a fluorescence LIVE/DEAD^®^ Viability/Cytotoxicity assay (Life Technologies, Austria) was used. Micrographs were taken by using an inverted fluorescence optical microscope (TE2000, Nikon, Japan) equipped with a digital camera (DS-Qi1MC, Nikon, Japan) using TRITC (Ex: 540/Em: 605) and GFP (Ex: 488/Em: 519) fluorescence bandpass filters.

### Compound Diffusion

HepG2 spheroids were incubated for 3 and 12 days and treated with rhodamine B (Roth, Germany) diluted to a final concentration of 100 µM in cell culture media. Fluorescence micrographs were taken every minute to monitor diffusion towards the spheroid core until fluorescence intensity profile of the spheroid reached the same level as fluorescence background. Intensity values of spheroid at 3 different depths were taken using ImageJ (NIH, USA) and monitored over time.

### Statistical and image analysis

Data were expressed as mean ± standard deviation (SD). All experiments were done independently in triplicates. Statistical significance among the experimental groups was determined with Student’s t-test. A P value < 0.05 was considered statistically significant (*). Graphs were plotted using Prism 6 (GraphPad software, USA). Micrographs were analyzed using ImageJ (NIH, USA). Spheroid cultivation was monitored over 18 days with an inverted fluorescence optical microscope (TE2000, Nikon, Japan) equipped with a digital camera (DS-Qi1MC, Nikon, Japan). Spheroid area, perimeter, solidity roundness and diameter were measured by using the BioVoxxel Image Processing and Analysis Toolbox for ImageJ (Biovoxxel, Germany). Drug penetration distances were determined by analyzing the RGB profile of fluorescence micrographs using ImageJ (see also Fig. S3).

## Results and Discussion

### HepG2 spheroid formation and cultivation

**Figure 1:**
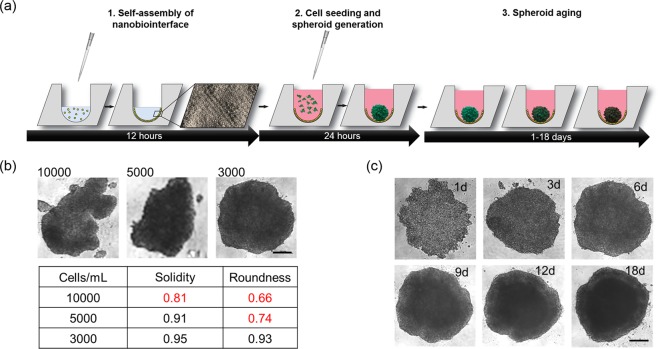
(**a**) Schematic workflow including time line of spheroid generation using self-assembled nanobiointerface. After seeding, cells form single multicellular spheroids for drug toxicity studies, based on different spheroid ages. (**b**) Phase-contrast optical micrographs show the impact of initial seeding density of HepG2 cells on spheroid solidity and roundness 6 days post-seeding. (**c**) Phase-contrast optical micrographs show the long-term evaluation of HepG2 spheroid shape at an initial seeding density of 3000 cells/well over a cultivation duration of 18 days. Scale bars, 200 µm.

Spheroid uniformity and quality including morphology, ultrastructure and organo-typic functionality are all crucial parameters and need to be optimized when using a novel protein-based cell-repulsive nanobiointerface for spheroid generation and long-term cultivation. Figure 1a shows the workflow used to generate the nanobiointerface inside the microtiter plate wells and generation of multi-cellular spheroids for drug toxicity measurements. Initial optimization investigates spheroid shape after 6 days in culture using increasing cell-seeding densities ranging from 3.000, 5.000 and 10.000 HepG2 cells per well. The dependence of spheroid shape on seeding density is illustrated in Fig. 1b where highest spheroid solidity and roundness (>0.9) is observed at a seeding density of 3.000 cells/well. Higher seeding densities resulted in inhomogeneous cell aggregates and unfavorable shape and size variations. Another important aspect of our dose-age relationship study is the long-term stability of HepG2 spheroids when cultivated over a period of 18 days on top of the self-assembled nanobiointerface. Results shown in Fig. 1c demonstrate that only in the presence of an initial seeding density of 3.000 cells per well no deformations in spheroid shape are obtained in long-term cultivations. As a result, the seeding density of 3.000 cells/well is chosen in the presence of our cell-repulsive protein nanobiointerface for all subsequent experiments.

### Evaluation of HepG2 spheroids based on morphology

**Figure 2:**
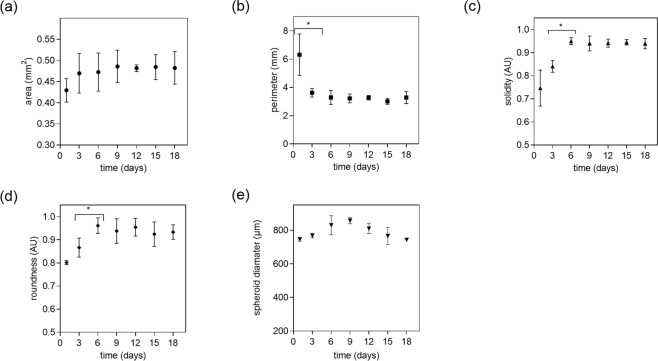
Influence of cultivation time on (**a**) area, (**b**) perimeter, (**c**) solidity (**d**) roundness and (**e**) diameter of HepG2 spheroids at a seeding density of 3000 cells/well. Error bars represent ± SD (n = 3) and *p < 0.05.

In a next set of experiments, additional key parameters of spheroid morphology including area, perimeter, solidity, roundness and diameter are monitored over a period of 18 days, as shown in Fig. 2.

At day 1 post-seeding individual HepG2 cells spontaneously self-assembled to an irregular shaped cell aggregate (see also Fig. 1c), exhibiting an area of 0.43 ± 0.027 mm^2^, a perimeter of 6.31 ± 3.18 mm, a solidity of 0.75 ± 0.25 and a roundness of 0.80 ± 0.01. At day 3 post-seeding spheroid area, solidity and roundness showed a slight increase, whereas a significant reduction in perimeter at the same time is observed (p < 0.05). The perimeter as an indicator for surface roughness decreased significantly within the first 3 days but remained constant over the remaining cultivation period. At day 6, a significant increase in spheroid solidity and roundness was noted, while perimeter and area remained at similar values (see Table S1). After day 6 no significant change in morphological parameters are evident (p > 0.05). Interestingly spheroid area did not increase significantly over time and remained in the range of 0.42 to 0.5 mm^2^. These results reveal that proper spheroid shape is reached at day 6 post-seeding and remained constant for a period of 3 weeks. This initial decrease in spheroid perimeter to 3.29 mm ± 0.5 mm and simultaneous increase of solidity to 0.95 ± 0.02 and a roundness of 0.93 ± 0.02 is consistent with literature on well-shaped spheroids exhibiting a roundness and a solidity above 0.9^31^. Spheroid diameter increased for the first 9 days from 746 ± 12 µm to 857 ± 19 µm and stayed stable for the following days of culture with 810 ± 30 µm for day 12, respectively. At day 15 and 18 spheroid diameter decreased to 766 ± 51 µm and 743 ± 10 µm indicating the start of spheroid disintegration. In the context of our dose-age relationship study, we therefore defined early-stage, mid-stage and late-stage HepG2 spheroids based on obtained differences in perimeter, solidity and roundness between days 3 to 5, 6 to 12 and 15 to 18, respectively.

### Histological and ultrastructural evaluation of HepG2 spheroid morphology

**Figure 3:**
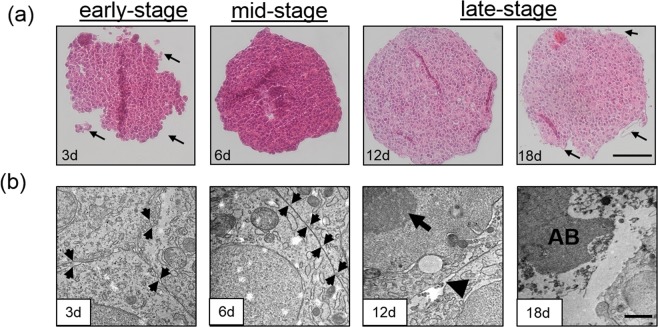
(**a**) Hematoxylin and eosin stained thin sections of early-stage spheroids at day 3 with loosely connected cell clusters (black arrows), mid-stage spheroids at day 6 with smooth spheroid surfaces and late-stage HepG2 spheroids starting at day 12 post-seeding with disintegrated spheroid surfaces (arrow). (**b**) Transmission electron microscopy micrographs of early-stage spheroids at day 3 with extracellular space between plasma membranes (black arrows), mid-stage spheroids at day 6 with tight junctions (black arrows) and late-stage HepG2 spheroids starting at day 12 post-seeding with blebbing of the cell surface (arrowhead), condensed chromatin (arrow) and apoptotic bodies (AB). Scale bar, 100 µm (top panel) and 1 µm (bottom panel).

A known drawback of aged spheroids is necrotic core formation, which is an unwanted phenomenon resulting from the accumulation of metabolic waste products and insufficient diffusion of oxygen/nutrients starting at a spheroid diameter above 200 to 500 µm^39,40^. To assess the overall structural architecture, HepG2 spheroid solidity and compactness was investigated in more detail using histochemistry and transmission electron microscopy (TEM). Histological and ultra-structural analysis as shown Fig. 3a,b demonstrates that early-stage HepG2 spheroids are loosely packed cell clusters interstitial spaces between individual cells, while mid-stage spheroids (day 6) display tight cellular junctions and an overall condensed spherical morphology with intact and smooth outer spheroid surfaces. Importantly, both early-stage and mid-stage spheroids revealed equally distributed chromatin in the nucleus as well as intact cytoplasm, thus indicating viable HepG2 cells. In contrast, late-stage spheroids at day 18 displayed typical apoptotic characteristics with loss of integrity of the outermost lining layer, specialized inter- and intra-cellular structures such as cell-cell contacts, shrinking of cytoplasm, membrane blebbing and formation of membrane-bound apoptotic bodies^41^. Nuclear shrinking and chromatin condensation, also referred as pyknosis, is observed in the cell nucleus and represents a hallmark of apoptosis^42^. Additionally, disintegration of the outmost surface was observable for late-stage spheroids. These results further confirm our above definition of late-staged spheroids between days 12 and 18 in culture where the formation of necrotic cores in late-stage apoptotic spheroids takes place (see also Fig. S1).

### Evaluation of secretion of organo-specific metabolites

**Figure 4:**
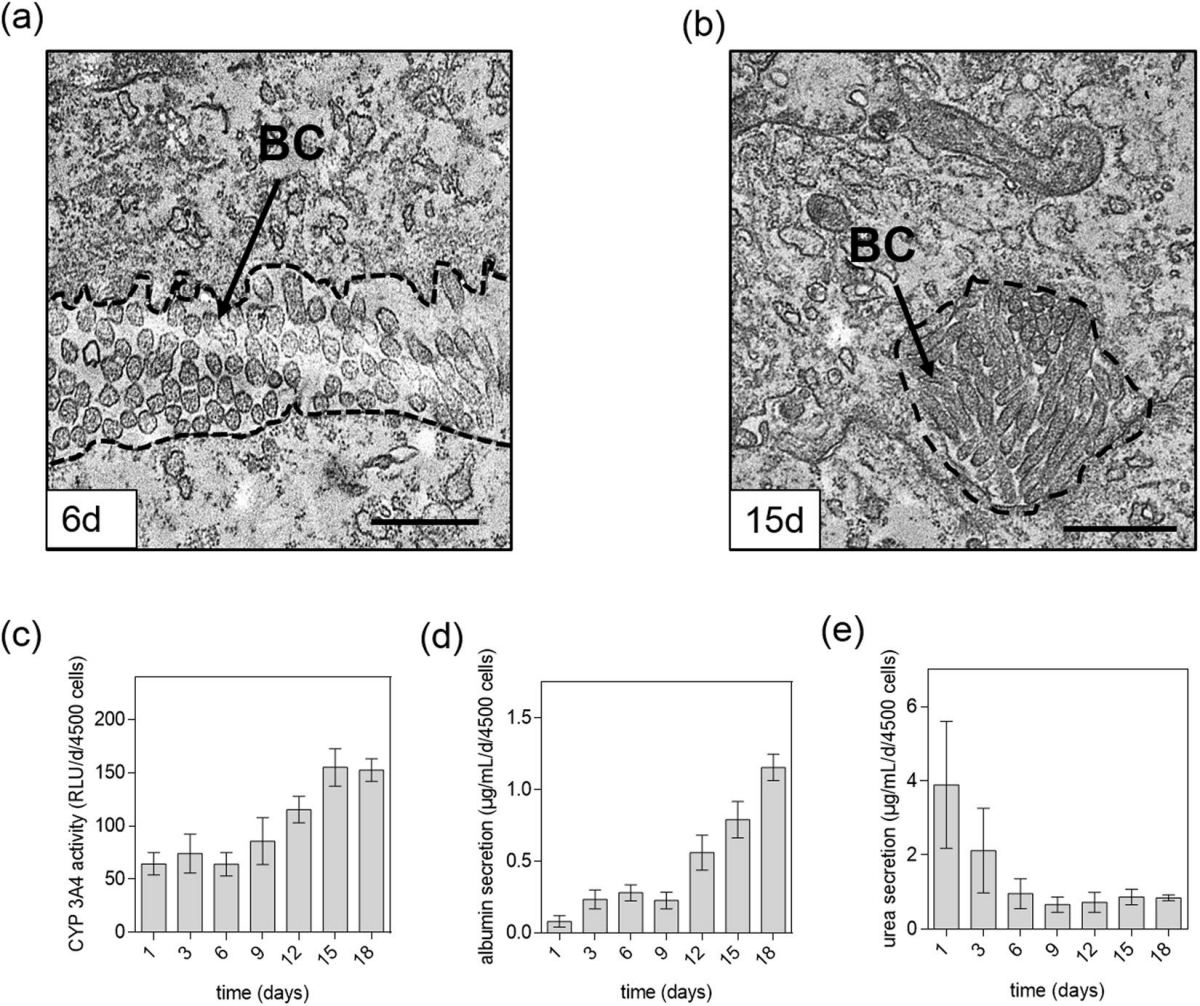
Transmission electron micrographs of HepG2 spheroids after (**a**) 6 days post-seeding and (**b**) 15 days post-seeding with organo-typic bile canaliculi (BC). Scale bars, 2 µm. (**c**) Activity of CYP 3A4 of HepG2 spheroids over time and secretion of (**d**) albumin and (**e**) urea of HepG2 spheroids over a cultivation period of 18 days. Error bars indicate ± SD (n = 3).

In addition to spheroid morphology, their organo-specific functionality is also an essential indicator for physiologically relevant 3D cell culture models. It is important to highlight that native liver tissue shows a highly specialized architecture and unique organization on a cellular level with highly specialized intercellular structures, so-called bile canaliculi, which are formed by plasma membranes of adjoining cells accounting for 15% of hepatocyte’s total plasma membrane surface^43^. To verify liver-specific ultrastructural intercellular morphology and metabolic functions of our HepG2 spheroids over an 18-day cultivation period, additional TEM measurements and metabolic assays were performed to assess the formation of bile canaliculi, albumin and urea secretion. Electron transmission microscopy results revealed that after day 6 post-seeding bile canaliculi with integrated luminal microvilli are present in mid and late-stage HepG2 spheroids as seen in Fig. 4a,b, while in early-stage spheroids only loosely associated cells are found that form softly packed aggregates lacking bile canaliculi. Both, mid-stage and late-stage spheroids displayed proper structural liver-specific phenotypes containing lumenized bile canaliculi that increase in diameter over time. In addition to the formation of specialized cellular structures, liver-specific metabolic activity including albumin secretion and urea excretion is evaluated in subsequent experiments. Since human CYP 3A4 has a major role in biotransformation and oxidation of bioactive compounds such as sorafenib^44^, the liver-specific enzymatic activity of CYP 3A4 was additionally monitored over incubation time of 18 days. As shown in Fig. 4c, CYP 3A4 activity remained constant for HepG2 spheroids until day 6 around 69 ± 5 RLU per day and 4500 cells. An increase of enzymatic activity was observable between day 6 to day 15 by 3-fold whereas values for HepG2 spheroids for day 18 did not further increase. In addition, Fig. 4d shows that initially, HepG2 spheroids secreted very low levels of albumin with 0.08 ± 0.1 µg/mL, 0.23 ± 0.07 µg/mL, 0.28 ± 0.06 µg/mL and 0.23 ± 0.05 µg/mL per day at day 1 to day 9, while gradual increase of albumin secretion is obtained with mid-stage and late-stage spheroids peaking at 1.15 ± 0.10 µg/mL per day at day 18. In contrast to albumin, urea concentration as shown in Fig. 4e in the collected supernatant samples decreased gradually over 18 days exhibiting an average excretion rate between 0.84 and 0.94 µg/mL per day and 4500 cells for mid-stage and late-stage spheroids. Even though tissue architecture may be changing for central zones of late-stage spheroids, these functional liver-specific evaluations suggest that between day 9 and 18 HepG2 spheroids display increased liver-specific activity compared to early stages.

**Figure 5:**
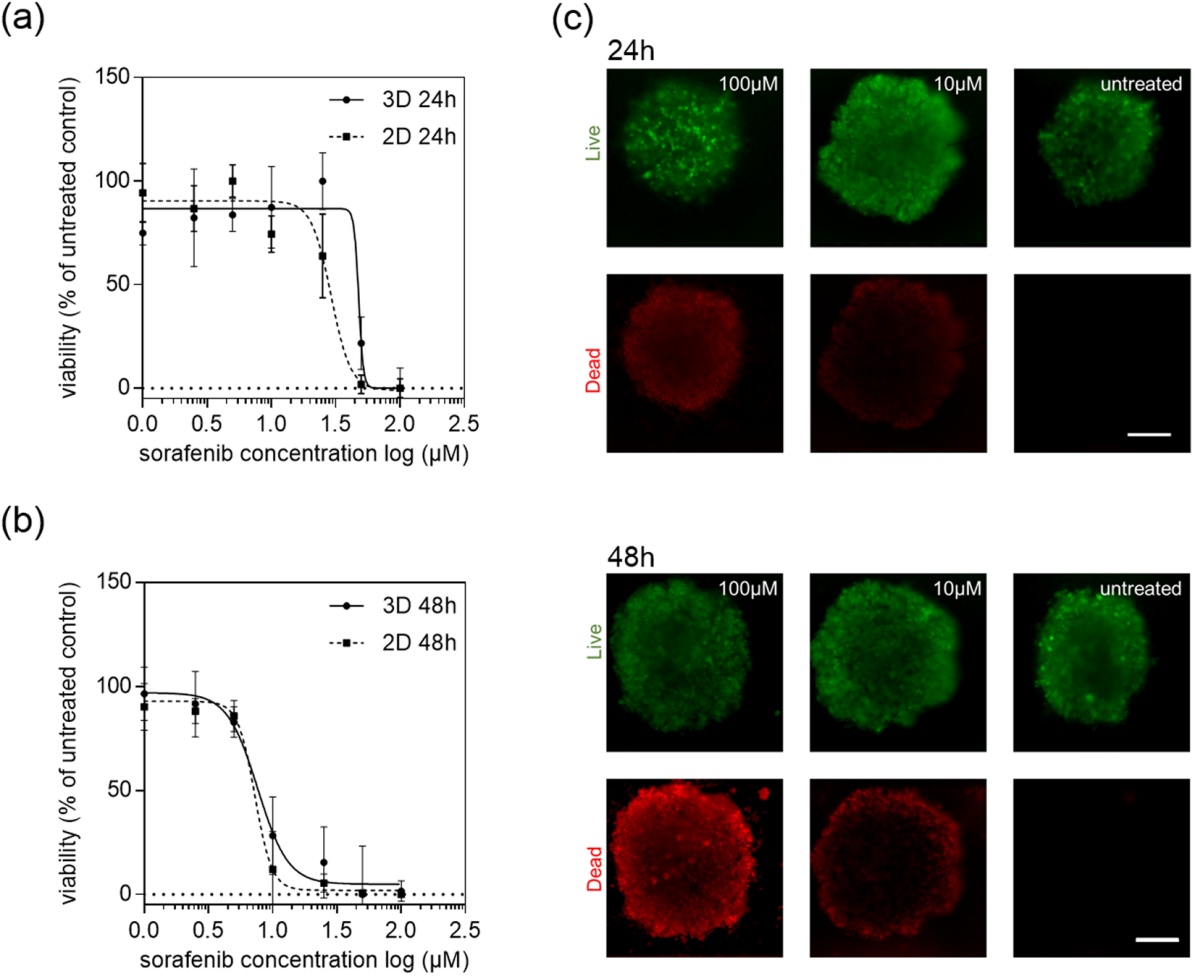
Sorafenib dose-response curves of HepG2 monolayers (2D) and spheroids (3D) after exposure time of (**a**) 24 hours and (**b**) 48 hours at day 6 post-seeding with (**c**) corresponding Live/Dead fluorescent micrographs. Error bars represent ± SD (n = 3).

Since our spheroid evaluation study also indicated that in the presence of an initial cell seeding density of 3.000 cells per well, HepG2 spheroids can be considered as organo-typic starting at day 6 to day 9 post-seeding, differences in dose-response relationships between mid-stage HepG2 spheroids and 2D monolayer culture are examined in follow-on experiments. Both 2D and 3D liver cell culture models  were exposed to increasing concentrations of sorafenib ranging from 0 µM to 100 µM for a period of 24 h and 48 h. Figure 5a shows the concentration-dependent inhibitory effects of sorafenib after 24 hours of drug exposure resulting in IC_50_ values for HepG2 spheroids and monolayer cultures of 47.77 ± 3.12 µM and 29.14 ± 1.14 µM, respectively. However, similar sorafenib dose-response curves are obtained for both HepG2 spheroids and 2D monocultures when exposure times are increased from 24 to 48 hours. Figure 5b shows cell viabilities in the presence of increasing sorafenib concentrations, exhibiting IC_50_ values 8.40 µM and 7.66 µM (p > 0.05) for spheroid and 2D monocultures, respectively. In other words, the required drug concentration necessary to inhibit 50% of the cells in HepG2 spheroids decreases from approximately 60 µM after 24 hours to 15 µM after 48 hours exposure, which translates to an overall 75% decrease. Following the same trend, an extended exposure time of 24 hours leads to a 95% decrease from 100 µM to 5 µM inhibitory concentration in the presence of 2D monolayer culture. Obtained IC_50_ values and p-values for both cell-culture methodologies are listed in Table 1 showing no statistical difference in growth inhibition after 48 hours. These results were also confirmed by Live/Dead viability assay based on Calcein AM and ethidium bromide as shown in Fig. 5c and compared to the dose-response behavior of HepG2 monolayer cultures (see Fig. S2 for Live/Dead images). This phenomenon can be attributed to several factors including (a) reduction of drug diffusivity into the spheroids, (b) the presence of quiescence cells and hypoxic areas within the core region of spheroids, (c) altered gene expression, (d) enhanced cell-cell contact and (e) the presence of ECM^45^.

**Table 1:** Half-maximal inhibitory concentration (IC_50_) of sorafenib for HepG2 spheroid (3D) and monolayer culture (2D) after 24 hours and 48 hours of drug exposure.

Sorafenib exposure time (hours)	3D (µM)	2D (µM)	p-value
24	47.77 ± 3.12	29.14 ± 1.14	<0.05
48	8.40 ± 1.20	7.66 ± 1.02	>0.05

**Figure 6:**
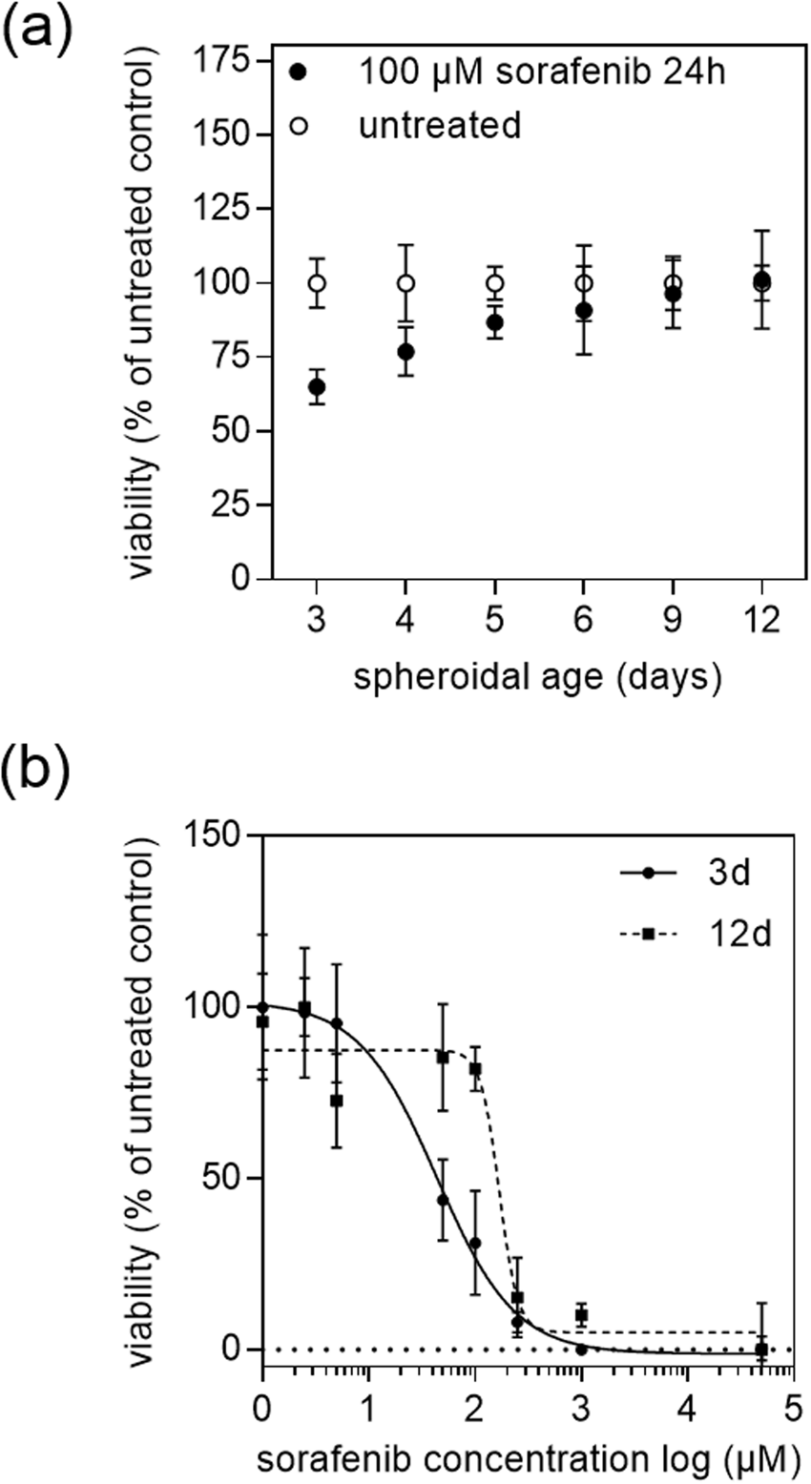
(**a**) Impact of spheroid age on viability of HepG2 spheroids after exposure to 100 µM sorafenib for 24 hours. (**b**) Sorafenib concentration-dependent response of 3 - and 12 days post-seeded HepG2 spheroids after an exposure period of 24 hours. Error bars represent ± SD (n = 3), *p < 0.05.

Next, the influence of spheroidal aging on drug diffusivity and resistance was investigated to gain a better understanding of the impact of culture time on drug efficacy. In a final set of experiments, early, mid-stage and late-stage HepG2 spheroids  were subjected to a 24-hour treatment of 100 µM sorafenib. Results based on time-resolved monitoring of cell viability are shown in Fig. 6a where sorafenib toxicity reduced the viability of our HepG2 spheroids to 65%, 77%, 86% at day 3, 4, and 5 post-seeding in comparison to untreated controls (overall relative standard deviation, RSD = 6%, n = 6). Surprisingly, already during the transition phase from early-stage spheroids (day 3) to mid-stage spheroids (day 5) showed an increase in drug resistance and reduced toxicity. After a cultivation period of 5 days, mid-stage HepG2 spheroids displayed no significant cytotoxic effect of sorafenib with viability values of 90.80%, 96.30% and 100.13% over the following 6 days (p > 0.05, overall RSD = 10%, n = 9). Interestingly, these results correlate well with the emergence of organo-typic microstructures and increased metabolic activities that start with the emergence of spheroid maturity around day 6. To verify the importance of spheroidal age on the outcome of drug toxicity studies, a comparative analysis of dose-response relationships of sorafenib between early (day 3) and late-stage (day 12) spheroids  were conducted in a final set of experiments. Figure 6b shows spheroid age-related dose-response curves obtained after 3 days and 12 days in culture resulting in elevated drug resistance to 100 µM sorafenib in late-stage spheroids.

Additionally, IC_50_ values listed in Table 2 show that late-stage HepG2 spheroids displayed elevated IC_50_ values of 168.70 ± 1.26 µM in comparison to early HepG2 spheroids of 43.75 ± 1.34 µM. This means when using late-stage spheroid a 4-fold higher concentration of sorafenib needs to be applied to reach approximately 50% inhibition of the cells compared to an early-stage HepG2 spheroid.

**Table 2:** Dose-age dependence of 3D HepG2 spheroids after 3 -and 12 days post-seeding.

Spheroidal age (days)	Mean IC_50_ value (µM)	SD
3	43.75	±1.34
12	168.7	±1.26

**Figure 7:**
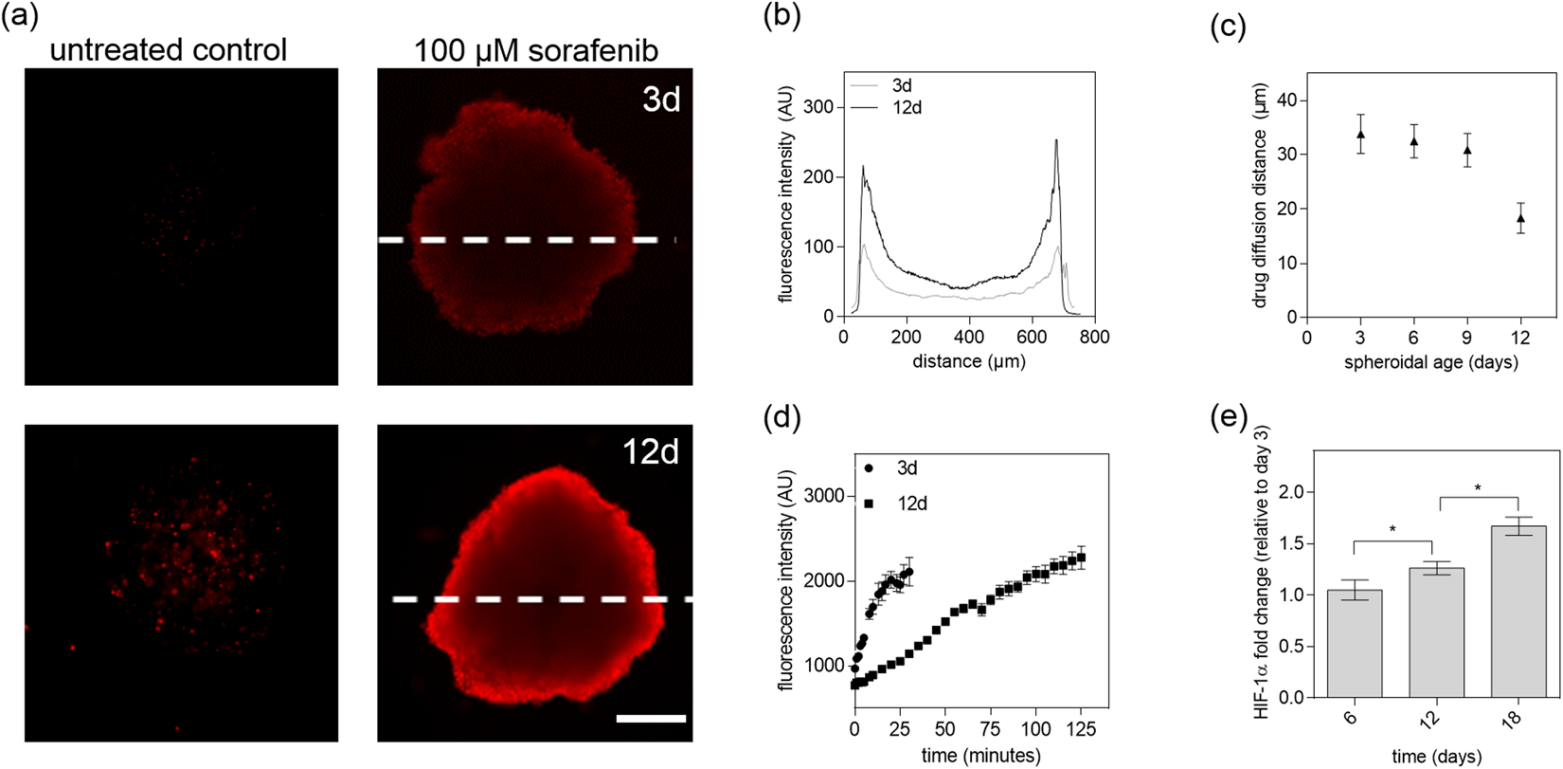
(**a**) Fluorescence images of early- (top panel) and late-stage (bottom panel) HepG2 spheroids with necrotic edges (red) after 24 hours of sorafenib exposure at a concentration of 0 µM and 100 µM. Scale bar, 200 µm. (**b**) Fluorescence intensity profile of early- and late-stage HepG2 spheroid images after treatment of 100 µM sorafenib for 24 hours. (**c**) Drug diffusion distance of sorafenib at a concentration of 100 µM after 24 hours exposure with respect to spheroidal age of HepG2 spheroids. Error bars represent ± SD (n = 4). (**d**) Inward diffusion of the fluorophore rhodamine B of early and late-stage HepG2 spheroids. (**e**) Fluorescence intensity fold change of HIF-1α immunohistochemical stained HepG2 spheroids relative to day 3 after cultivation time of 6, 12 and 18 days post-seeding. Error bars represent ± SD (n = 4) and *p < 0.05.

To investigate this age-related effect in more detail, drug penetration depth of sorafenib was analyzed using a fluorescent dye-exclusion assay (e.g. Live/Dead cytotoxicity assay). Results from our drug penetration study show the formation of an apoptotic outer rim at a sorafenib concentration of 100 µM for early and late-stage HepG2 spheroids as seen in Fig. 7a. The observed apoptotic edge is caused by the strong cytotoxic effect of the drug on the outer-most cell layers since fluorescent intensity profile analysis of spheroid cross-sections shows highest intensities of dead cells (red channel for necrotic cells) near the outer rim of the spheroid and decreases gradually towards the spheroid core. Late-stage spheroids exhibit an even higher intensity at the rim caused by the more compact outer cell layers of otherwise properly formed spherical structures. However, when calculating the drug diffusion distance as a ratio between living and dead cells a significant decrease in diffusion distance from around 30 ± 3 µm in mid-stage spheroids (up to day 9) to 18 ± 2 µm for late-stage spheroids (after day 12 post seeding) is evident as shown in Fig. 7b. To quantify inward diffusion distance in more detail, rhodamine B was chosen as a model molecule that features molecular weight like sorafenib. As shown in Fig. 7d, early stage HepG2 spheroids display a 4.2-fold higher diffusivity towards molecules of molecular weight around 470–480 g/mol already plateauing after 30 and 125 minutes in the central core region of HepG2 spheroids respectively. In addition, further histochemical evaluation of central spheroid regions confirmed that HIF-1 positive hypoxic cells were gradually increasing with 100% hypoxia-positive cells at day 18 post-seeding (see Fig 7e and S4). Overall these results point at different penetration depths of bioactive compounds between early, mid-stage and late-stage spheroids, due to changes of organotypic architecture and function of HepG2 spheroids. This limited drug diffusion into the inner regions in aged tumor models significantly decreases the efficacy of chemotherapeutic drugs.

## Conclusion

*In vitro* multi-cellular spheroid models have become a promising tool in drug discovery and development, but comparability and reproducibility remain a pressing issue. Additionally, the lack of standardization, scalability, and compatibility with current screening systems has led to large lab-to-lab discrepancies and inconsistent data outcome in drug studies even when using the same tissue type^46^. As a consequence, the reproducible generation of multi-cellular spheroids including shape, size, cell density and morphology, is key in increasing the reliability of *in vitro* 3D-cell based assays. In the present work, we have investigated the impact of spheroidal age on drug efficacy to gain a deeper understanding how methodological inconstancies influence the outcome of drug screening studies.

Using a protein-based nanobiointerface, we were able to establish HepG2 spheroids of similar size, shape and morphologies, while structural evaluation showed the formation of liver-specific morphologies over an 18-day cultivation period. Based on our ultrastructural and organo-typic functional investigations, distinctly different spheroid phases were identified including an early (day 3 to 6), mid-stage (day 6 to 12) and late stage (day 15 to 18) development. The three spheroidal development stages show significant differences in cell-to-cell interactions, specialized microstructures such as bile canaliculi formation, and metabolic activists including albumin and urea secretion. Results from our initial spheroidal aging study revealed a decreased sorafenib toxicity of following a 24-hour exposure with early, mid-stage and late-stage HepG2 spheroids. In fact, a 4-times higher sorafenib doses are needed to exhibit similar toxic effects in late-stage spheroids when compared to early-stage spheroids. To investigate this age-related effect in more detail, drug penetration depths were analyzed resulting in a significant decrease in diffusion distance from 31 ± 3 µm in mid-stage spheroids (up to day 9) to 18 ± 3 µm for late-stage spheroids. This phenomenon can be explained through intercellular interactions and physical limitations such as higher interstitial fluid pressure, which is known to restrict drug transport into deeper regions of the spheroid and thus leads to enhanced resistance to chemotherapeutics^33^. Additionally, increasing cell densities as natural diffusion barriers as well as biological limitations of complex *in vitro* models (e.g. apoptosis due to limited membranes permeability, an increasing acidic microenvironment and hypoxia in the central spheroid core regions)^47^ have been linked to reduced efficacy of radio -and chemotherapies^48,49^. Independent of the reasons for the observed drug transport limitations, our study has clearly demonstrated that spheroidal age needs to be considered as an important variable for future drug sensitivity tests using spheroid-based *in vitro* models.

## Supplementary information

suppl info
