# Supplementary material for: Effect of Spheroidal Age on Sorafenib Diffusivity and Toxicity in a 3D HepG2 Spheroid Model
Source: Sci Rep. 2019 Mar 19;9:4863. doi: 10.1038/s41598-019-41273-3 (PMC6425026; doi:10.1038/s41598-019-41273-3)
Supplement: Supplementary file 1 — suppl info [file 41598_2019_41273_MOESM1_ESM.docx]

Supplementary Information

# Effect of Spheroidal Age on Sorafenib Diffusivity and Toxicity in a 3D HepG2 Spheroid Model

Christoph Eilenberger ^a,b^, Mario Rothbauer ^a,*^, Eva-Kathrin Ehmoser ^b^, Peter Ertl ^a,*^ and Seta Küpcü^b^

*^a^ Institute of Applied Synthetic Chemistry and Institute of Chemical Technologies and Analytics, Faculty of Technical Chemistry, Vienna University of Technology, Getreidemarkt 9, 1060 Vienna, Austria*

*^b^ Institute of Synthetic Bioarchitectures, Department of Nanobiotechnology, University of Natural Resources and Life Sciences, Vienna, Muthgasse 11, 1190 Vienna, Austria*

**corresponding authors:* [*mario.rothbauer@tuwien.ac.at*](mailto:mario.rothbauer@tuwien.ac.at)*,* [*peter.ertl@tuwien.ac.at*](mailto:peter.ertl@tuwien.ac.at)

**Keywords:** HepG2 Tumor Spheroids, S-Layer, Spheroidal Age, Sorafenib, Drug Resistance, Drug Diffusivity

**Supplementary Table 1. Spheroid Morphology**

**Supplementary Table 1** HepG2 spheroid shape evolution over time at an initial seeding density of 3000 cells per well. After day 6 post-seeding the cell aggregates can be considered as compact spherical spheroids (>0.9). Values above 0.9 are displayed in red.

| Day | Area (mm^2^) | Perimeter (mm) | Solidity | Roundness |
| --- | --- | --- | --- | --- |
| 1 | 0.43 | 6.31 | 0.75 | 0.80 |
| 3 | 0.47 | 3.62 | 0.84 | 0.87 |
| 6 | 0.47 | 3.29 | 0.95 | 0.93 |
| 9 | 0.49 | 3.22 | 0.94 | 0.94 |
| 12 | 0.48 | 3.28 | 0.94 | 0.95 |
| 18 | 0.48 | 3.28 | 0.94 | 0.93 |

**Supplementary Figure 1. Spheroid ultrastructure**

**
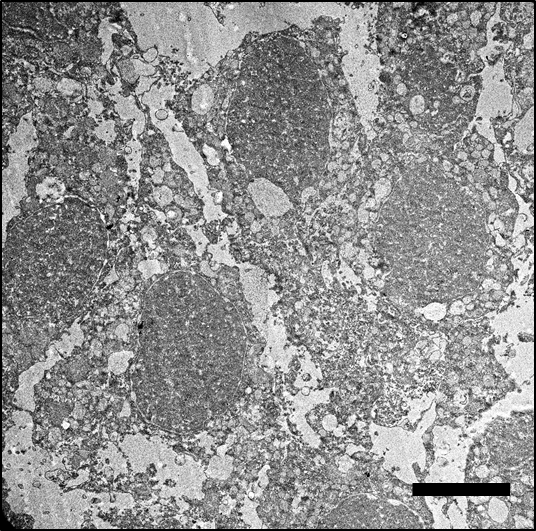
**

**Figure** **S1** Transmission electron microscopy micrograph of late-stage HepG2 spheroids at day 18 post-seeding with extruded cell fragments, indicating apoptosis. Based on this observation, very late-stage spheroids were considered as apoptotic and not taken into account for following age-drug response experiments. Scale bar, 5 µm.

**Supplementary Figure 2: Viability of sorafenib treated HepG2 monolayers**

**
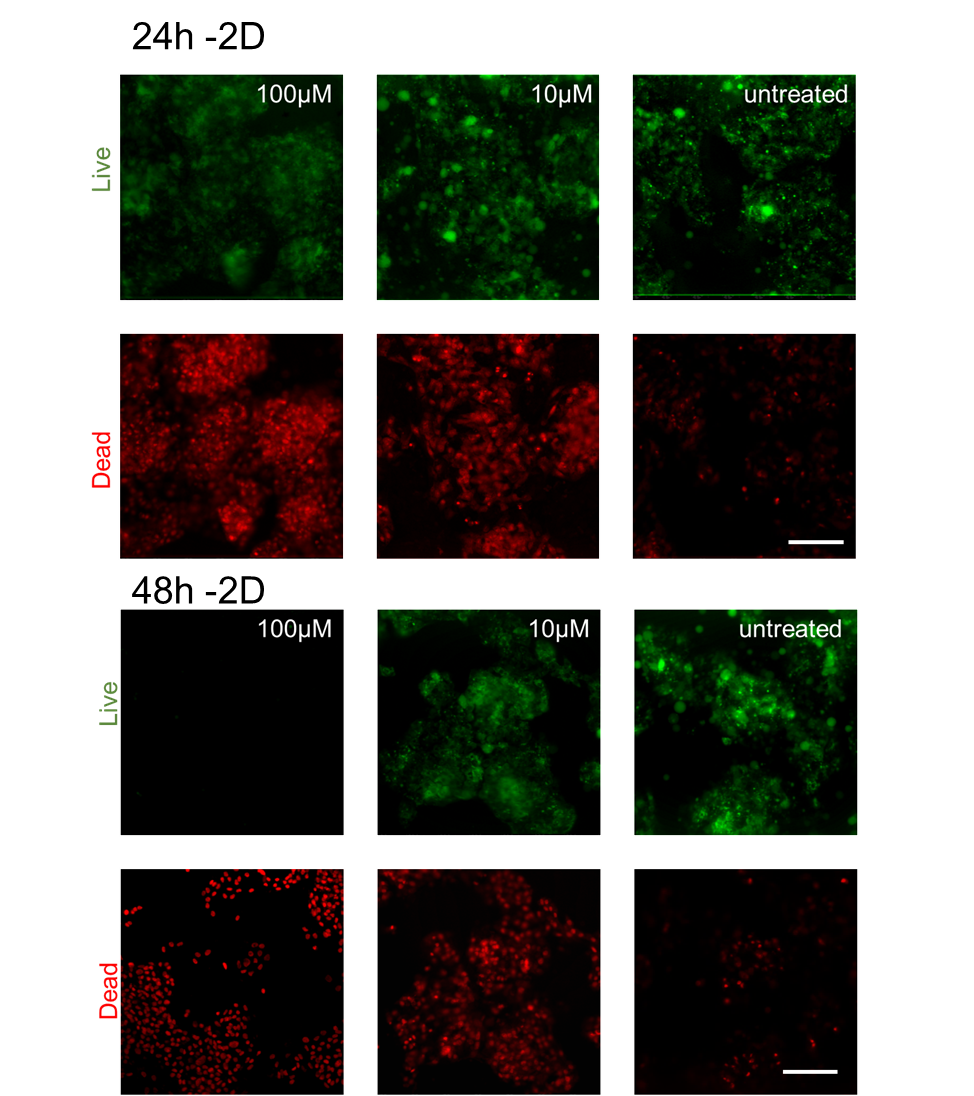
**

**Figure S2** Live/Dead staining of HepG2 monolayer after exposure time of 24 and 48 hours with 100 µM, 10 µM and 0 µM sorafenib at day 6 post-seeding. Scale bars, 200 µm.

**Supplementary Figure 3: Sorafenib drug diffusion**

**
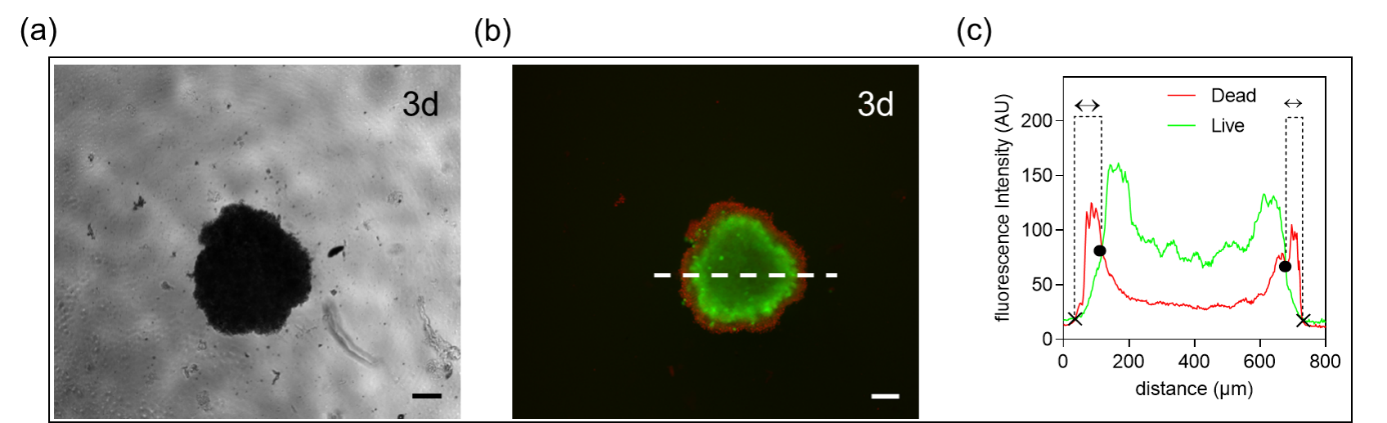
**

**Figure S3 (a)** Bright field and **(b)** fluorescence micrographs of a live (green)–dead (red) assay on HepG2 spheroids after 3 days of incubation. Scale bar, 200 µm. **(c)** The RGB profiles for both channels show the distribution of live and dead cells in the HepG2 spheroid. The drug diffusion distances (dotted line) were calculated by measuring the length of the red fluorescence signal that overlaps the green signal at the outer rim (between • and x ), indicating inward diffusion of the cytotoxic drug sorafenib.

**Supplementary Figure 4: Immunohistochemical detection of hypoxia-inducible-factor-1α**


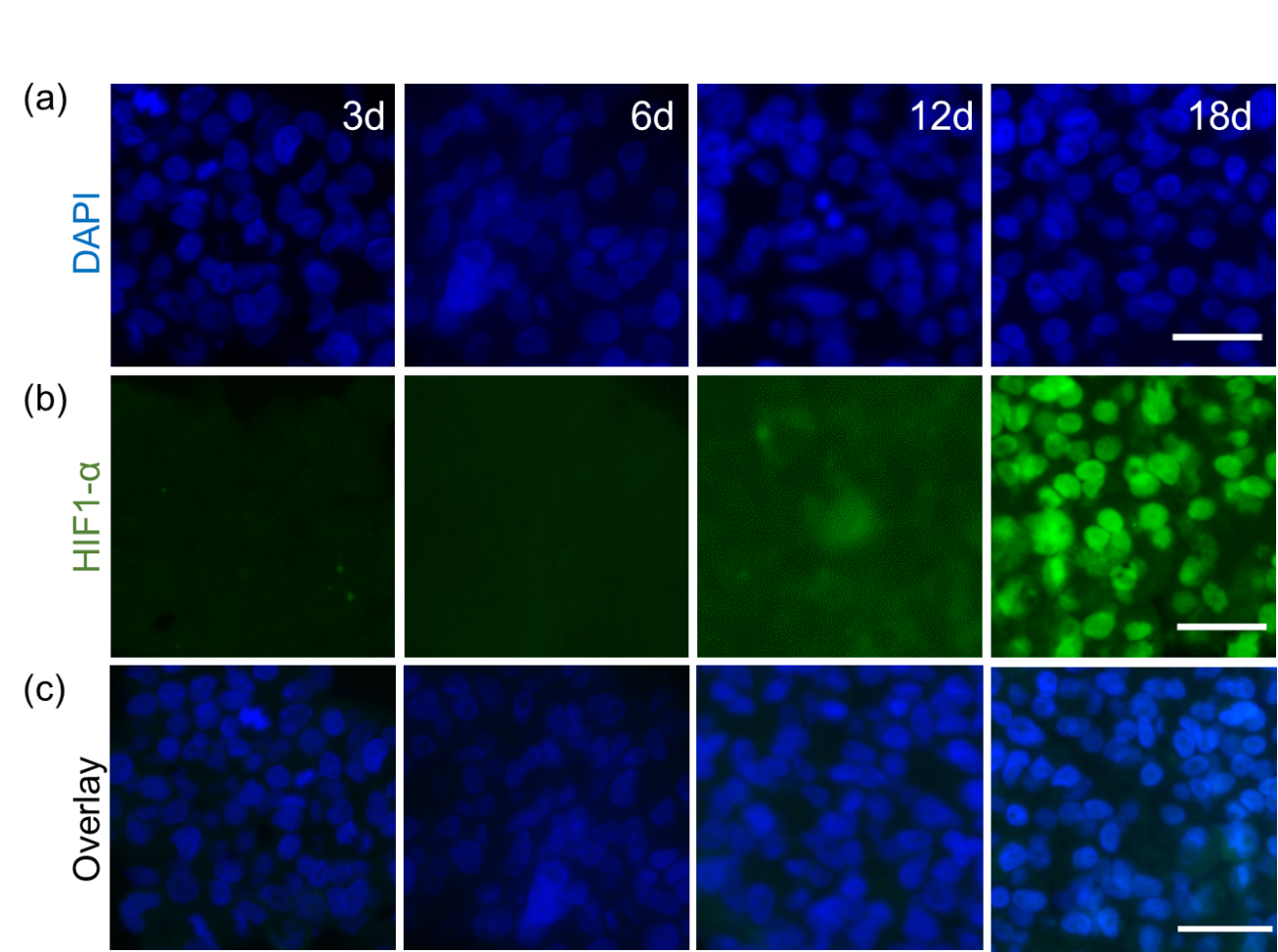


**Figure S4** Hypoxic core formation of HepG2 spheroids over a cultivation time of 18 days illustrated by **(a)** fluorescence stained cell nuclei, **(b)** immunohistochemical staining with anti-HIF1-α mAb and **(c)** an overlay of both channels. Scale bar, 20 µm.
